# LRP1 is critical for the surface distribution and internalization of the NR2B NMDA receptor subtype

**DOI:** 10.1186/1750-1326-8-25

**Published:** 2013-07-17

**Authors:** Wladislaw Maier, Mariola Bednorz, Sabrina Meister, Anton Roebroek, Sascha Weggen, Ulrich Schmitt, Claus U Pietrzik

**Affiliations:** 1 University Medical Center of the Johannes Gutenberg-University of Mainz Institute of Pathobiochemistry, Duesbergweg 6, Mainz 55099, Germany; 2 Department of Psychiatry and Psychotherapy, University Medical Center of the Johannes Gutenberg-University of Mainz, Untere Zahlbacher Str. 8, Mainz 55131, Germany; 3 Laboratory for Experimental Mouse Genetics, Center for Human Genetics KU Leuven, Herestraat 49, Leuven B-3000, Belgium; 4 Department of Neuropathology Molecular Neuropathology, Heinrich Heine University Duesseldorf, Moorenstr. 5, Duesseldorf, 40225, Germany

**Keywords:** LRP1, NPxY2 motif, NMDA receptor, NR1, NR2B receptor subunit, PSD95, Cell surface expression

## Abstract

**Background:**

The N-methyl-D-aspartate receptors are key mediators of excitatory transmission and are implicated in many forms of synaptic plasticity. These receptors are heterotetrameres consisting of two obligatory NR1 and two regulatory subunits, usually NR2A or NR2B. The NR2B subunits are abundant in the early postnatal brain, while the NR2A/NR2B ratio increases during early postnatal development. This shift is driven by NMDA receptor activity. A functional interplay of the Low Density Lipoprotein Receptor Related Protein 1 (LRP1) NMDA receptor has already been reported. Such abilities as interaction of LRP1 with NMDA receptor subunits or its important role in tPa-mediated NMDA receptor signaling were already demonstrated. Moreover, mice harboring a conditional neuronal *knock-out* mutation of the entire *Lrp1* gene display NMDA-associated behavioral changes. However, the exact role of LRP1 on NMDA receptor function remains still elusive.

**Results:**

To provide a mechanistic explanation for such effects we investigated whether an inactivating *knock-in* mutation into the NPxY2 motif of LRP1 might influence the cell surface expression of LRP1 and NMDA receptors in primary cortical neurons. Here we demonstrate that a *knock-in* into the NPxY2 motif of LRP1 results in an increased surface expression of LRP1 and NR2B NMDA receptor subunit due to reduced endocytosis rates of LRP1 and the NR2B subunit in primary neurons derived from LRP1ΔNPxY2 animals. Furthermore, we demonstrate an altered phosphorylation pattern of S1480 and Y1472 in the NR2B subunit at the surface of LRP1ΔNPxY2 neurons, while the respective kinases Fyn and casein kinase II are not differently regulated compared with wild type controls. Performing co-immunoprecipitation experiments we demonstrate that binding of LRP1 to NR2B might be linked by PSD95, is phosphorylation dependent and this regulation mechanism is impaired in LRP1ΔNPxY2 neurons. Finally, we demonstrate hyperactivity and changes in spatial and reversal learning in LRP1ΔNPxY2 mice, confirming the mechanistic interaction in a physiological readout.

**Conclusions:**

In summary, our data demonstrate that LRP1 plays a critical role in the regulation of NR2B expression at the cell surface and may provide a mechanistic explanation for the behavioral abnormalities detected in neuronal LRP1 *knock-out* animals reported earlier.

## Background

Low density lipoprotein receptor-related protein 1 (LRP1) is a member of the lipoprotein receptor family. LRP1 is synthesized as a 600 kDa full-length precursor molecule in the endoplasmic reticulum and subsequently cleaved by furin in the Golgi network generating an 85 kDa transmembrane β-subunit that remains noncovalently associated to the extracellular 515 kDa α-subunit. The α-subunit contains four ligand binding domains for more than 30 ligands while the β-subunit contains two intracellular NPxY motifs [1]. Generation of cells from mice carrying a *knock-in* mutation in the NPxYxxL (NPxY2) sequence revealed a reduced internalization rate for LRP1 [2-4]. The NPxY2 motif has been shown to interact with the majority of known intracellular interaction partners of LRP1, many of which only bound the tyrosine phosphorylated form [5,6]. Beyond its role as a cargo receptor LRP1 has been frequently associated with cell signaling events and has been demonstrated as an important element in NMDA receptor signaling [7,8]. Functional NMDA receptors are heterotetrameres formed by two obligatory NR1 subunits and two regulatory NR2 subunits, while NR1 is encoded by a single gene the NR2 subunits are derived from four unique genes (NR2A-D) [9-11]. The NR2B receptor subunits are abundant in early postnatal brain and in young neurons in culture (days *in vitro* (DIV) 9 to 15), while the expression of NR2A subunits increases with development [12-16]. This shift in NR2A/NR2B ratio seems to be driven at least in part by sensory experience and, therefore, by NMDA receptor activity [14,16,17]. The physiological role of these changes may be the optimization of the threshold for inducing synaptic activity at different developmental points. Different regulation mechanisms of NR2A and NR2B subunits regarding their synthesis, trafficking, degradation and surface expression have been described [18]. The surface expression of NMDA receptors is a tightly regulated process in response to e.g. phosphorylation events induced by ligand-binding and during the synapse maturation [19]. The serine phosphorylation within ESDV motif of NR2B (S1480) by casein kinase II (CKII) leads to an increased endocytosis rate of the NR2B subunit [20,21]. However, tyrosine phosphorylation within the YEKL motif of NR2B (Y1472) by Fyn kinase [22] has been demonstrated to inhibit the internalization of the NR2B subunit [23,24]. Nevertheless, the experiments performed using mutant NR2B subunits, carrying a point mutation at Y1472, demonstrated that the receptor endocytosis was not completely blocked in these mutants [25], indicating additional regulation mechanisms for internalization.

Since LRP1 has been implicated in NMDA receptor function, we investigated in this study whether an inactivating *knock-in* mutation of the NPxY2 motif in the endogenous gene for LRP1 shows a direct influence on NMDA receptor function and subsequently NMDA receptor mediated learning and memory phenotypes. We demonstrate that a *knock-in* into the NPxY2 motif of LRP1 leads to its reduced endocytosis in primary neurons and concomitantly results in an increased surface deposition of NR2B and NR1 receptor subunits in primary cortical neurons. Furthermore, we demonstrate that the accumulation of NR2B at the cell surface of LRP1ΔNPxY2 neurons resist the regulation through phosphorylation within YEKL and ESDL motifs of NR2B. Additionally, we demonstrate that the interaction of NR2B and LRP1 might be linked by PSD95, is phosphorylation dependent and its regulation requires a functional NPxY2 motif of LRP1. Based on these molecular data we were able to demonstrate NMDA-associated behavioral changes in LRP1ΔNPxY2 mice. In summary, our data demonstrate that LRP1 plays a critical role in the regulation of NR2B expression at the cell surface and may provide a mechanistic explanation for the behavioral abnormalities detected in LRP1 *knock-out* animals reported earlier [26,27].

## Results

### Functional knock-in into the NPxY2 motif of LRP1 leads to an altered expression of NMDA receptor subunits at the surface of primary neurons

Although various aspects of the influence of LRP1 expression on NMDA receptor signaling have been studied, the exact mechanism of NMDA receptor regulation by LRP1 is still elusive [8,26,27]. To unravel this mechanism we analyzed whether the C-terminal domain of LRP1 might influence NMDA receptor function. Therefore we used primary cortical neurons of mice harboring a *knock-in* mutation in the NPxYxxL motif of LRP1 (LRP1ΔNPxY2) [3]. Our results provide first evidence for a direct influence of LRP1 on the cell surface expression and the internalization regulation of NMDA receptor subunits and demonstrate that a *knock-in* into NPxY2 motif of LRP1 finally leads to changes in learning and memory.

**Figure 1:**
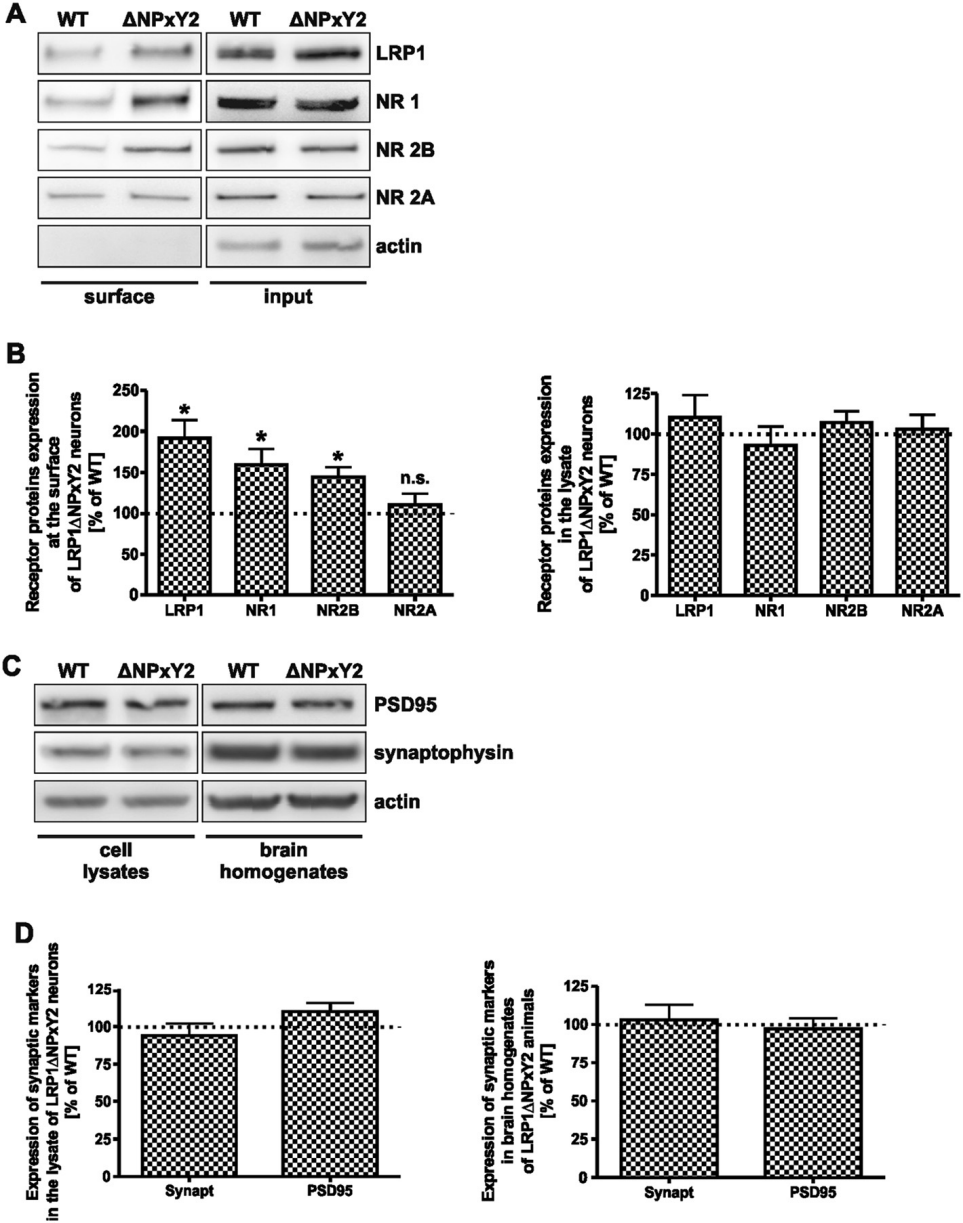
**LRP1 and NMDA receptor subunits NR1/NR2B are increased at the surface of cortical LRP1ΔNPxY2 neurons. (A)** Representative immunoblots demonstrate an increase in surface expression of LRP1 and NMDA receptor subunits NR1/NR2B in primary LRP1ΔNPxY2 neurons. The surface expression of NR2A is not altered. Primary cortical WT or LRP1ΔNPxY2 neurons were subjected to cell surface biotinylation prior to lysis. Biotinylated proteins were precipitated with NeutrAvidin agarose and analyzed by SDS-PAGE and Western blot (*surface*). As input controls 15µg of entire cell lysates proteins were used (*input*). **(B)** The protein expression at the cell surface was quantified by densitometric analysis. The intensities of the surface signals were normalized to the intensities of lysate signals (*input*). The calculated values for WT were set as 100%. The scale bars represent the mean percent change in the expression of proteins at surface of LRP1ΔNPxY2 neurons compared with WT controls ± S.E.M. for the expression of LRP1 at the surface of LRP1ΔNPxY2 neurons an increase by approx. 90% (p=0.01; n=4); for NR1 and NR2B an increase by approx. 60% (p=0.007; n=4) and by approx. 44 % (p=0.04; n=4) respectively. The signal intensities of lysates were standardized to the signal intensities of actin. * p<0.05, Student´s paired *t*-test. **(C)** Representative immunoblots demonstrate unaltered expression rates of synaptophysin and PSD95 in neuronal cell lysates or in brain homogenates of LRP1ΔNPxY2 or WT animals. **(D)** The densitometric analysis of multiple Western blots demonstrated any significant alterations in expression of synaptophysin or PSD95 in cell lysates or brain homogenates of LRP1ΔNPxY2 mice. The scale bars represent the mean percent change in the expression of synaptophysin or PSD95 in neuronal lysates or brain homogenates derived from LRP1ΔNPxY2 animals compared with WT controls ± S.E.M. The signal intensities of lysates were standardized to the signal intensities of actin. n=5.

Previously it was shown that internalization of LRP1 is altered in mouse embryonic fibroblasts harboring a *knock-in* mutation in the distal NPxY motif of LRP1 [2,4,28]. Accordingly, we extended our analysis to primary cortical neurons (DIV14) derived from LRP1ΔNPxY2 mice or from wild type controls, employing the surface biotinylation technique. We observed an approximate 90% (p = 0.01, n = 4) increase in LRP1 surface expression in LRP1ΔNPxY2 neurons compared with wild type controls, while the *steady state* expression of the protein in the cell lysates was unaltered (Figure 1A, upper panels; Figure 1B). A recent published study demonstrated a decreased expression of NMDA receptor subunit NR1 in the brain homogenates of the animals harboring a forebrain LRP1 *knock-out* mutation [27]. Additionally, several studies have already demonstrated that the cell surface expression of NMDA receptors is a prerequisite for normal NMDA receptor activity [24,29,30]. Taken together these data indicate a role of LRP1 in the regulation of the expression or cell distribution of NMDA receptor subunits. According to the data presented by Liu and colleagues earlier, we tested the expression of NMDA receptor subunits in the cell lysates of primary cortical neurons or whole brain homogenates derived from LRP1ΔNPxY2 animals or wild type controls [27]. We were not able to detect any significant alterations in the expression rates of NMDA receptor subunits NR1, NR2B or NR2A in the cell lysates of primary cortical LRP1ΔNPxY2 neurons (Figure 1A right panels, Figure 1B) or in brain homogenates derived from LRP1ΔNPxY2 or wild type animals (data not shown). To test whether the LRP1ΔNPxY2 *knock-in* mutation influences the cellular distribution of NMDA receptors we performed surface biotinylation experiments in LRP1ΔNPxY2 and LRP1 wild type neurons. Interestingly, the surface expression of NR1 and NR2B receptor subunits in cortical neurons derived of LRP1ΔNPxY2 mice was increased by 60% (p = 0.007, n = 4) and by 44% (p = 0.04, n = 4) respectively, while the surface expression of the NR2A receptor subunit was unaltered compared with wild type controls (Figure 1A left panels, Figure 1B). Previously a drastic reduction in the expression of synaptic markers synaptophysin and PSD95 in the brain homogenates of animals harboring a forebrain *knock-out* mutation of the entire LRP1 gene has been reported [27]. According to these results we investigated the *steady state* expression of synaptophysin and PSD95 in the brain homogenates and cell lysates of primary neurons derived from LRP1ΔNPxY2 animals or wild type controls. In contrast to the results obtained from full LRP1 *knock-out* animals we were not able to detect any significant changes in the expression of PSD95 or synaptophysin in neuronal cell lysates or brain homogenates of LRP1ΔNPxY2 animals compared to the wild type controls (Figure 1C,D). Furthermore, the cell viability of primary cortical neurons derived from LRP1ΔNPxY2 animals compared to wild type controls employing an AlamarBlue assay (Invitrogen) or measurement of neurite outgrowth demonstrated no significant differences between the genotypes (Additional file 1: Figure S1A and 1B and Additional file 2).

### Reduced internalization of LRP1 results in enhanced surface expression of NR1/NR2B receptor subunits

**Figure 2:**
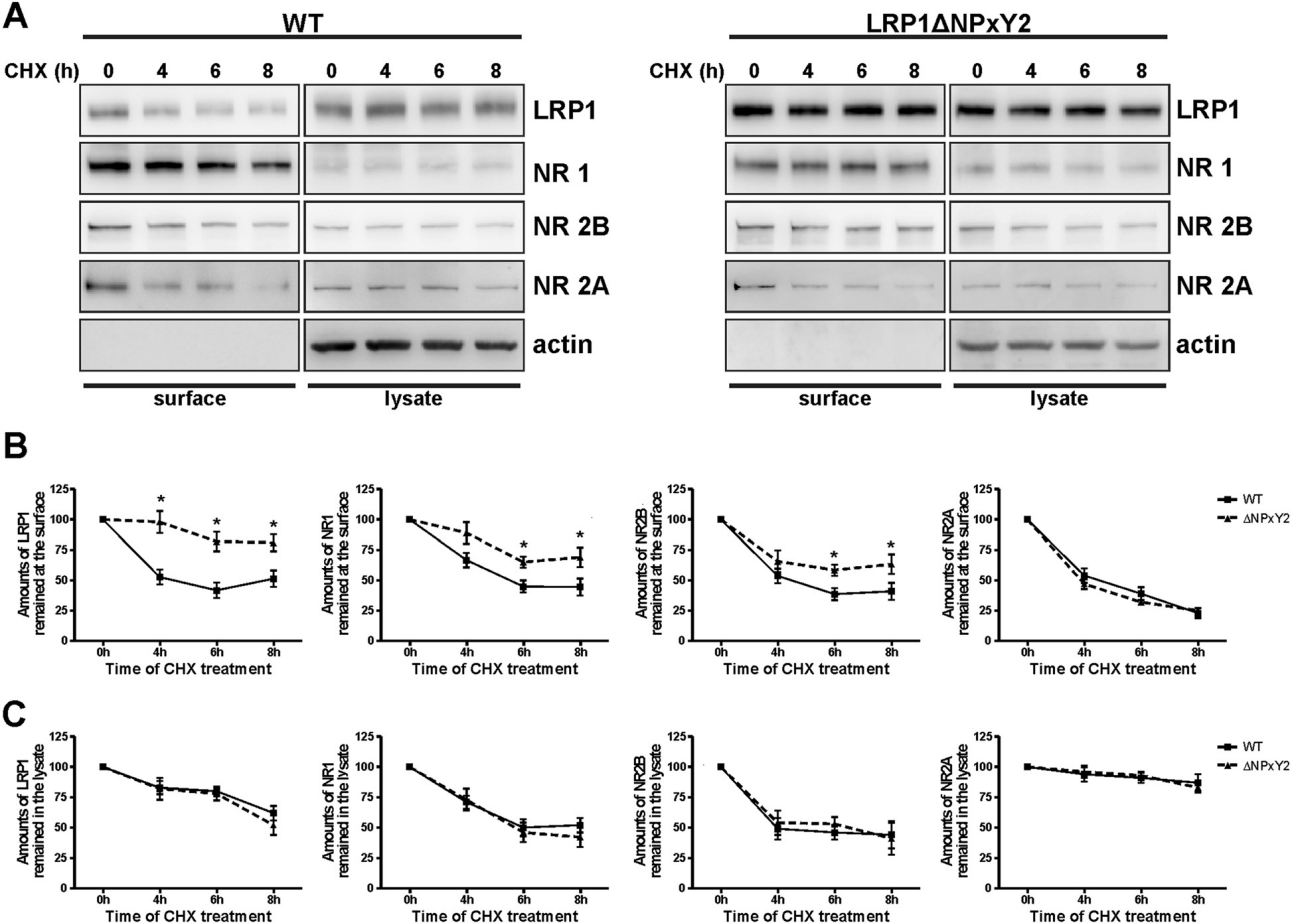
**Surface expression of LRP1 and NR1/NR2B is increased in LRP1ΔNPxY2 neurons after treatment with cycloheximide. (A)** Immunoblots show an increase in residual expression of LRP1 and NR1/NR2B receptors at the cell surface of LRP1ΔNPxY2 neurons after treatment with CHX. The primary neurons (DIV14) were treated with 20µg/ml CHX for 4, 6 and 8 hours and subjected to cell surface biotinylation. Biotinylated proteins were precipitated with NeutrAvidin agarose (*surface*). **(B)** The immunoblots were quantified by densitometric analysis. The intensities of the surface signals were normalized to the intensities for lysate signals (*input*). The values for the 4h, 6h and 8h time points were compared with respective values for 0h time point. The values for 0h time points were set as 100%. The diagrams represent the mean percent change in the residual expression of proteins at the surface of LRP1ΔNPxY2 or WT neurons after treatment with 20µg/ml CHX ± S.E.M. For the residual expression of LRP1 at the cell surface of LRP1ΔNPxY2 neurons an increase by 89% (p=0.03; n=4) after 4h, by 94% (p=0.02; n=6) after 6h and by 59% (p=0.043; n=4) after 8h was calculated. For the residual expression of NR1 by 33% (p=0.073; n=4) after 4h; by 58% (p=0.03; n=6) after 6h and by 67% (p=0.042; n=5) after 8h. For the expression of NR2B an increase by 22% (p=0.06; n=4) after 4h; by 51% (p=0.032; n=6) after 6h and by 54% (p=0.04; n=4) after 8h. The residual expression of NR2A was not significantly altered in LRP1ΔNPxY2 neurons. **(C)** The degradation rates of investigated proteins are not altered in LRP1ΔNPxY2 neurons. The diagrams represent the mean percent change in the expression of proteins in lysates of LRP1ΔNPxY2 or WT neurons after CHX-treatment ± S.E.M. The signal intensities of lysates were standardized to actin. * p<0.05, Student´s paired *t*-test.

The increase in surface expression of LRP1 and the NMDA receptor subunits NR1 and NR2B raised the question, whether the altered surface expression is due to a faster transport of these proteins to the cell surface, a reduced degradation rate or rather an effect of an impaired internalization of the respective proteins. We used a cycloheximide (CHX)-mediated protein degradation assay to address this question. CHX is a protein translation inhibiting agent. Since CHX treatment will reduce general protein translation and subsequently reduce the amount of newly synthesized proteins at the cell surface, we hypothesized that proteins showing reduced endocytosis will accumulate at the cell surface. We were able to demonstrate that LRP1 surface expression in LRP1ΔNPxY2 neurons was increased after CHX treatment for different time periods, indicating that reduced internalization is responsible for this surface accumulation (Figure 2A,B). This assumption was further supported by the observation that no significant differences in the degradation rates of respective proteins in the cell lysates during the CHX treatments were observed as demonstrated in Figures 2A and 2C. Most interestingly, the NR1 and NR2B subunits showed a similar increase in surface expression in LRP1ΔNPxY2 neurons after CHX treatment with the strongest effect after 6 h of incubation (Figure 2A,B). So we were able to determine a 94% (p = 0.02; n = 6) increase in residual LRP1 expression at the surface of LRP1ΔNPxY2 neurons after 6 h CHX treatment (Figure 2B), while the residual amounts of NR1 and NR2B receptor proteins were increased by approximately 58% (p = 0.03; n = 6) and 51% (p = 0.032; n = 6) respectively compared to wild type controls (Figure 2B). From these results we concluded that the enhanced surface expression of NMDA receptor subunits and LRP1 is rather an effect of a slower internalization of these receptors in LRP1ΔNPxY2 neurons, than an effect of a faster transport to the cell surface or a reduced protein degradation rate in LRP1ΔNPxY2 neurons.

**Figure 3:**
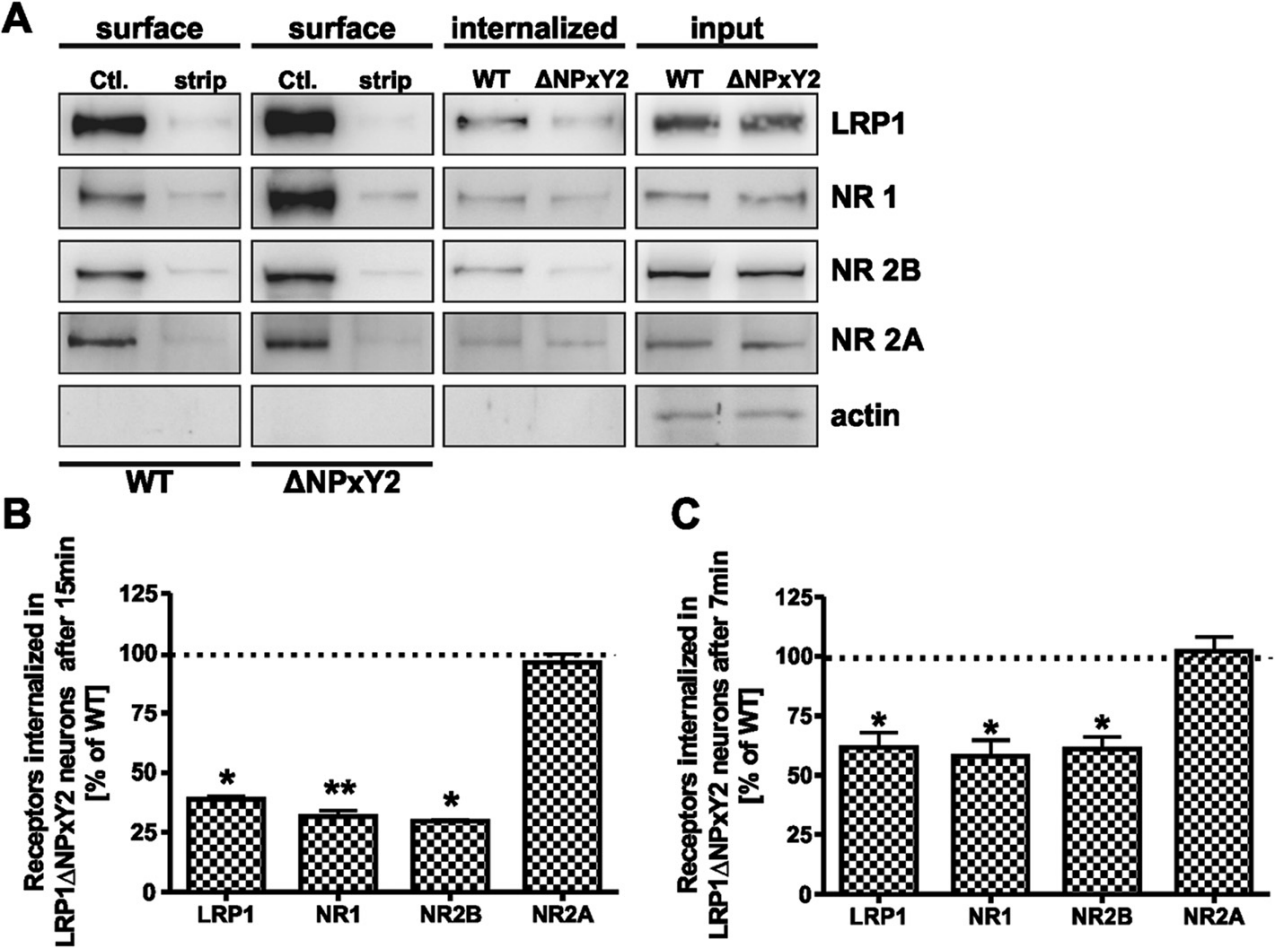
**LRP1 and NR1/NR2B accumulate at cell surface of LRP1ΔNPxY2 neurons due to reduced endocytosis. (A)** The Internalization rates of LRP1 and NMDAR were determined using a cleavable biotin assay in WT or LRP1ΔNPxY2 neurons. Two left panels (*surface*) demonstrate that MesNa buffer removes cleavable NHS-SS-biotin from the cell surface. Middle panels (*internalized*) show the amounts of internalized proteins after 15min at 37°C. Right panels (*input*) show the total amounts of proteins. Demonstrated blots represent 15min period of internalization. **(B)** The immunoblots were quantified by densitometric analysis. The internalization rates were calculated by comparing the values of internalized biotinylated proteins (*internalized*) to the values of respective biotinylated proteins at the cell surface of neurons in control dishes (*Ctl.*) and normalized to the signal intensities of the total amounts of the proteins (*input*). The internalization rates calculated for WT controls were set as 100%. The scale bars represent the mean percent change in internalization rates of proteins at the surface of LRP1ΔNPxY2 neurons compared with WT controls ± S.E.M. For the internalization rate calculated after 15min of internalization in LRP1ΔNPxY2 neurons for LRP1 a reduction by approx. 60% (p=0.01; n=5); for NR1 and NR2B a decrease by approx. 69% (p=0.001; n=5) and by approx. 70% (p=0.016; n=5) respectively. For the internalization rate of NR2A an insignificant decrease by 4% (n=4) in LRP1ΔNPxY2 neurons was calculated. **(C)** The internalization rates calculated in LRP1ΔNPxY2 neurons after 7 min of internalization. For LRP1 a reduction by 38% (p=0.043; n=4); for NR1 and NR2B a reduction by approx. 42% (p=0.04; n=4) and by approx. 39% (p= 0.044; n=5) was determined. For the internalization rate of NR2A an insignificant increase by 2% (n=4) in LRP1ΔNPxY2 neurons was calculated. The signal intensities of lysates were standardized to actin. * p<0.05; ** p<0.005 Student´s paired *t*-test.

To further support the data derived from the CHX-degradation assay we directly measured the internalization rates of cell surface proteins, performing cleavable biotin internalization experiments. In this assay the surface proteins were labeled with cleavable NHS-SS Biotin at 4°C to prevent the internalization. Subsequently, the neurons were incubated at 37°C for 7 min or 15 min to initiate internalization. The internalization process was halted by putting the cells back to 4°C and the remaining cell surface bound biotin was cleaved off by treating the cells with MesNa buffer. Therefore the biotinylated proteins detected in cell lysates represent the fraction of proteins which were internalized from the cell surface. As we have proposed before, we were able to detect a reduction of the internalization rate by 60% for LRP1 (p = 0.01, n = 6), by 69% for NR1 (p = 0.001, n = 6) and by 70% for NR2B (p = 0.02, n = 5) in LRP1ΔNPxY2 neurons compared with the wild type controls after 15 min (Figures 3A and 3B). The investigation of the internalization rates of NR2A in LRP1ΔNPxY2 or wild type neurons revealed no significant alterations (Figure 3). An alternative explanation for the demonstrated decrease in the internalization rates of LRP1 and NR1/NR2B receptors in LRP1ΔNPxY2 neurons might be an accelerated recycling of these receptors at the cell surface. To test this hypothesis we analyzed the internalization rates after a shorter time period of 7 min. As demonstrated in Figure 3C we detected a reduction of the internalization rates for LRP1 by 38% (p = 0.043; n = 4), for NR1 by 42% (p = 0.04; n = 4) and for NR2B by 39% (p = 0.044; n = 5) after internalization period of 7 min. Similar to the internalization rates measured after 15 min these results revealed a similar tendency for the reduction in the internalization rates of LRP1 and NR1/NR2B receptors in LRP1ΔNPxY2 neurons. This indicates that the observed increase in the expression of these proteins at the cell surface and the reduced internalization rates are probably not an effect of accelerated recycling events in LRP1ΔNPxY2 neurons. In summary, we observed an increase in surface expression of LRP1 and the NMDA receptor subunits NR1 and NR2B, but not NR2A in LRP1ΔNPxY2 neurons which is an effect of a reduced internalization of these receptors due to the inactivating *knock-in* mutation in the endogenous *Lrp1* gene.

### The LRP1ΔNPxY2 knock-in substitution leads to an altered phosphorylation pattern of the NR2B receptor subunit

**Figure 4:**
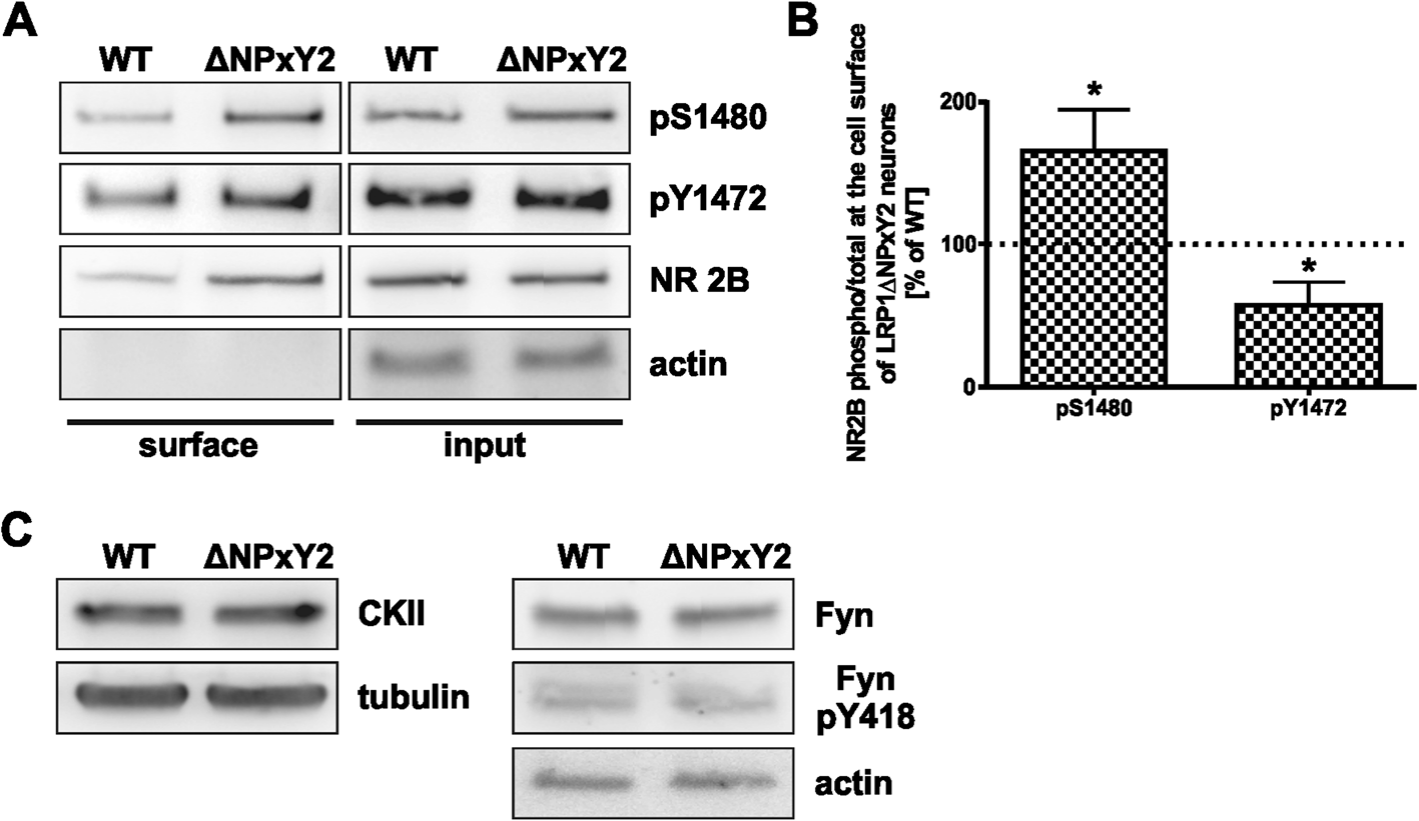
**LRP1ΔNPxY2 neurons show an altered phosphorylation of S1480 and Y1472 in NR2B receptor subunit. (A)** Representative immunoblots demonstrate altered amounts of phosphorylated S1480 and Y1472 in NR2B receptor subunit at the surface of LRP1ΔNPxY2 neurons. Cortical LRP1ΔNPxY2 or WT neurons (DIV14) were subjected to the cell surface biotinylation. Biotinylated proteins were precipitated with NeutrAvidin agarose (*surface*). Membranes were probed with phospho-specific polyclonal NR2BpS1480 or NR2BpY1472 antibodies. Parallel, the same amounts of biotinylated proteins and input controls were loaded on the same SDS-Gel. The respective immunoblot was analyzed with a monoclonal NR2B antibody, to detect the entire amounts of biotinylated NR2B receptor at the cell surface for normalization of phospho-signals. **(B)** Protein expression was quantified by densitometric analysis of multiple blots. The intensities of cell surface signals were normalized to measured signal intensities for lysate controls (*input*). The obtained values for phosphorylated S1480 or Y1472 were normalized to signal intensities for the entire cell surface expression of NR2B receptor subunit. The values calculated for WT controls were set as 100%. The scale bars represent the mean percent change in the phosphorylation on S1480 or Y1472 of NR2B receptor subunit at surface of LRP1ΔNPxY2 neurons compared with WT controls ± S.E.M. for the phosphorylation of S1480 of LRP1ΔNPxY2 neurons an increase by 64% (p=0.04; n=4) and for the phosphorylation of Y1472 a decrease to 66% (p=0.03; n=4) of WT controls after a normalization to the entire NR2B cell surface expression. * p<0.05, Student´s paired *t*-test. **(C)** Representative Western blots demonstrate that the expression of casein kinase II or Fyn is not altered in LRP1ΔNPxY2 neurons. The phosphorylation levels of Fyn activation signal pY418 are not altered in LRP1ΔNPxY2 neurons. The membranes were probed with a phospho-specific polyclonal anti-Src pY418 antibody, which also reacts with Fyn pY418.

So far we were able to demonstrate a reduced internalization of NR1/NR2B receptor subunits in cortical neurons of LRP1ΔNPxY2 *knock-in* mice. However, the molecular mechanism of LRP1 mediated NMDA receptor subunit endocytosis is still elusive. Recently a regulation mechanism for the surface distribution of NR2B receptor subunit has been suggested [21]. The authors investigated the neuronal maturation-dependent switch from the NR2B subunit to the NR2A receptor subunit at the surface of cortical neurons. In this context two phosphorylation sites in the NR2B amino acid sequence were described as regulatory signals for the NR2B surface distribution [21]. Phosphorylation at tyrosine Y1472 within the YEKL C-terminal motif of NR2B has been demonstrated to stabilize the receptor subunit at the cell surface by inhibiting the binding to AP2, an adaptor protein for clathrin-mediated endocytosis [23]. In contrary, the phosphorylation at serine S1480 within the ESDV motif of NR2B has been shown to interrupt the interaction with the PDZ domain of PSD95 and to trigger the Y1472 dephosphorylation leading to endocytosis of NR2B [20]. Consequently the phosphorylation at these positions might serve as regulatory signals for the surface expression of the NR2B subunit [23,25]. Based on these observations, we investigated the phosphorylation pattern of the NR2B receptor subunit at the cell surface of primary neurons derived from LRP1ΔNPxY2 mice or wild type controls. Interestingly, we observed an increase in the phosphorylation of S1480 by approximately 64% (p = 0.04, n = 4) in NR2B subunits at the cell surface in neurons carrying the LRP1ΔNPxY2 *knock-in* mutation (Figure 4A; upper panel). However, the densitometric analysis of multiple western blots revealed a reduction in phosphorylation of Y1472 to approximately 66% (p = 0.03, n = 4) compared with wild type controls (Figure 4A middle panel, Figure 4B). The position S1480 has been demonstrated to be a substrate for casein kinase II (CKII), while the phosphorylation at position Y1472 has been shown to be facilitated by Fyn kinase [20,22]. An altered expression or activation of respective kinases in LRP1ΔNPxY2 neurons might be a possible explanation for the observed phosphorylation pattern of NR2B receptor subunit. Therefore we investigated the expression rate and the activation status of CKII and Fyn in the neuronal lysates derived from LRP1ΔNPxY2 neurons or wild type controls. As demonstrated in Figure 4C we were not able to detect any differences in the expression rates of CKII or Fyn in LRP1ΔNPxY2 neurons. Furthermore, we were unable to detect an increased phosphorylation at position Y418 of Fyn in LRP1ΔNPxY2 neurons which has been shown to be the activation signal of the kinase (Figure 4C) [31]. Therefore, the modified phosphorylation pattern of the NR2B subunits at the surface of primary LRP1ΔNPxY2 neurons is not an effect of altered expression or activity of respective kinases. Nevertheless, the observed phosphorylation pattern of NR2B subunits at the cell surface of LRP1ΔNPxY2 neurons indicates a stronger activation of the internalization signals for NR2B, while its accumulation at the cell surface of LRP1ΔNPxY2 neurons seems to overcome these regulation mechanisms.

### Knock-in mutation in NPxY2 motif of LRP1 results in a stronger binding of NR2B to LRP1

**Figure 5:**
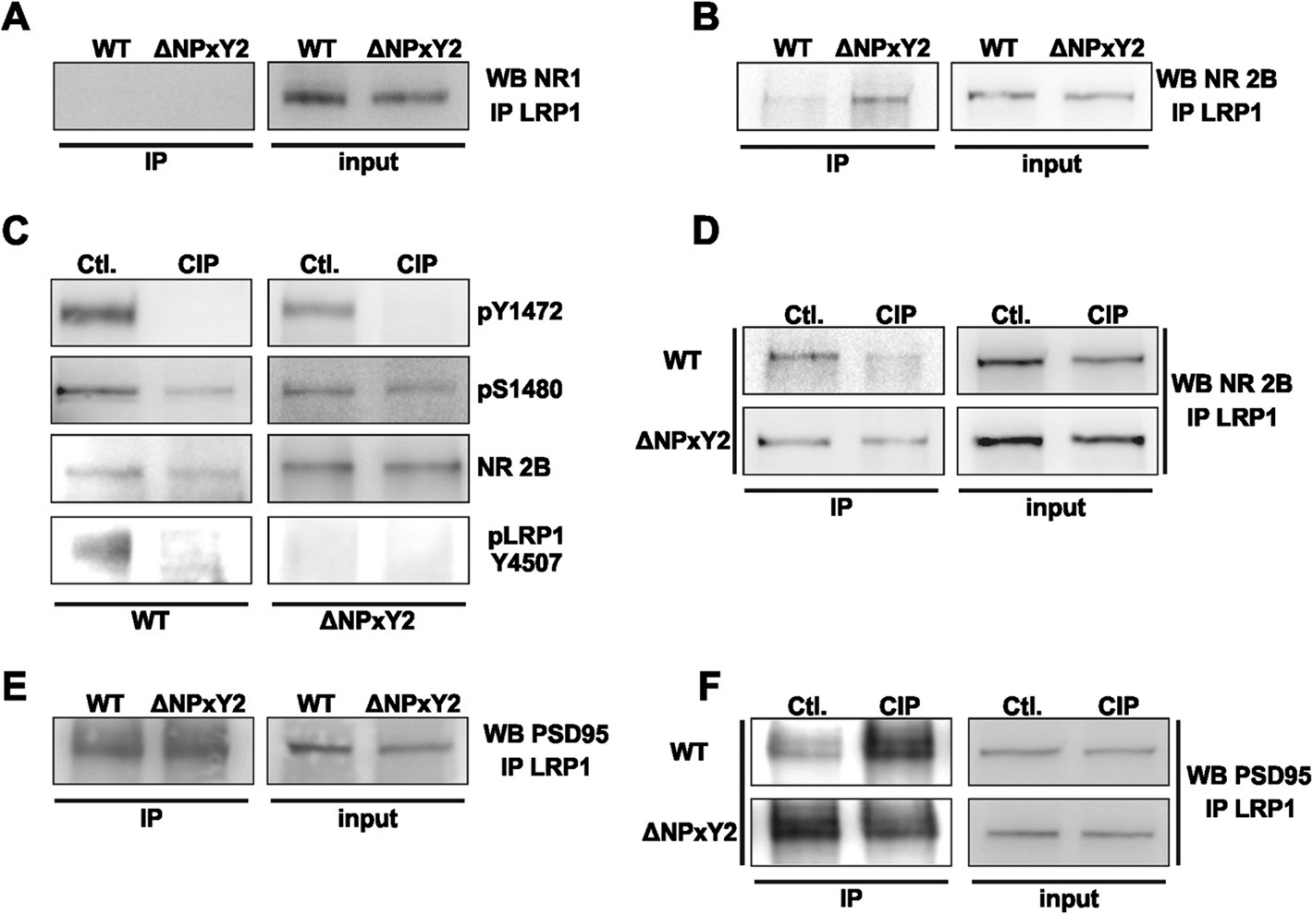
**Knock-in mutation into NPxY2 motif of LRP1 results in stronger binding of NR2B with LRP1. (A)** The representative immunoblot demonstrates the lacking of interaction of NR1 with LRP1 in a co-immunoprecipitation experiment. Equal amounts of total lysates (80µg) were used for immunoprecipitation with a polyclonal LRP1-specific antibody (1704) [50]. The membranes were probed with a monoclonal NR1-specific antibody. **(B)** The representative immunoblot demonstrates a stronger interaction of NR2B with LRP1 in cortical LRP1ΔNPxY2 neurons. After an immunoprecipitation with a LRP1-specific antibody (1704) [50] the membranes were probed with a monoclonal NR2B-specific antibody. **(C)** A treatment with CIP results in a complete dephosphorylation of tyrosines Y4507 in wild type LRP1 and Y1472 in NR2B, while serine 1480 in NR2B shows a residual phosphorylation. The equal amounts of CIP-treated lysates and untreated controls were analyzed. Membranes were probed with a polyclonal phospho-specific LRP1pY4507 antibody and polyclonal phospho-specific antibodies NR2BpY1472 or NR2BpS1480. **(D)** The dephosphorylation of proteins in cell lysates of WT neurons results in a reduction of NR2B co-immunoprecipitated with LRP1, while a dephosphorylation of LRP1ΔNPxY2 lysates has no effect on the binding of NR2B to LRP1. CIP-treated neuronal lysates derived of WT or LRP1ΔNPxY2 cortical cultures or untreated controls were used for co-immunoprecipitation. The co-purified proteins were detected with a monoclonal NR2B-specific antibody. **(E)** The binding of PSD95 with LRP1 is not directly affected by LRP1ΔNPxY2 *knock-in* mutation. Representative immunoblot demonstrates the equal amounts of PSD95 protein co-precipitated with LRP1 in cell lysates of LRP1ΔNPxY2 neurons or WT controls. **(F)** The dephosphorylation of proteins in cell lysates of WT neurons results in an increase of PSD95 co-immunoprecipitated with LRP1, while a dephosphorylation of proteins in LRP1ΔNPxY2 lysates has no effect on the binding of PSD95 to LRP1. Immunoblots shown are representative results from at least three independent experiments.

Our results presented so far indicate a direct role of LRP1 on the internalization process of NMDA receptor subunits NR1 and NR2B. Since we and others have already demonstrated a functional interplay of LRP1 with NMDA receptor subunits [8,26], we investigated the direct interaction of LRP1 with NR1 or NR2B subunits in neurons derived from wild type or LRP1ΔNPxY2 *knock-in* mice. We were not able to demonstrate an interaction of LRP1 with NMDA receptor subunit NR1 in co-immunoprecipitation experiments as shown in Figure 5A. This goes along the lines of published data showing no interaction between LRP1 and the NR1 subunit [26]. Most interestingly, we observed an increased amount of NR2B receptor protein co-precipitated with LRP1 in the lysates of LRP1ΔNPxY2 neurons (Figure 5B). These results indicate a stronger interaction of LRP1 with NR2B receptor subunit in LRP1ΔNPxY2 neurons compared to wild type controls (Figure 5B). It has been demonstrated that C-terminal interactions of LRP1 or NR2B subunits with cytosolic proteins are phosphorylation dependent [5,6,23,25]. To analyze whether the phosphorylation status of LRP1 or the NR2B subunit might influence their binding properties, we performed a calf intestinal phosphatase (CIP) dephosphorylation assay followed by co-immunoprecipitation [32]. We treated neuronal cell lysates with CIP (10 units/μg protein) for 30 minutes at 37°C. CIP activity in cell lysates and dephosphorylation of LRP1 were verified with a phospho-specific LRP1 pY4507 antibody (Figure 5C, lower panels). Since the position Y4507 does not exist in the LRP1 amino acid sequence of LRP1ΔNPXY2 mice we were unable to detect any phosphorylation in the presence or absence of CIP (Figure 5C lower panel). To proof the dephosphorylation of the NR2B subunit we performed immunoblot analysis with a phospho-specific antibodies and demonstrated a complete tyrosine dephosphorylation at position Y1472 (Figure 5C upper panel). However, a residual phosphorylation at position S1480 in the NR2B subunit after CIP treatment could be detected (Figure 5C middle panels). After validation of the CIP dephosphorylation in both receptors we investigated whether these changes might influence the direct binding of the NR2B receptor subunit to wild type LRP1 or mutated LRP1ΔNPxY2 [32]. We were able to demonstrate a drastically reduced amount of NR2B receptor protein co-immunoprecipitated with LRP1 in CIP-treated wild type neuronal lysates (Figure 5D upper panel). However, the dephosphorylation of the proteins in neuronal lysates derived of LRP1ΔNPXY2 neurons had no effect on the binding of NR2B with LRP1ΔNPxY2 (Figure 5D, lower panel). Previously we demonstrated that an over expression of PDZ domains or of a full length PSD95 construct resulted in a decrease of LRP1-mediated Erk1/2 activation by NMDA receptors. Furthermore, we demonstrated that the NPxY2 motif of LRP1 is important for NMDA receptor-mediated signal transduction [8]. According to these results we tested whether PSD95 interacts with LRP1 in co-immunoprecipitation experiments. As demonstrated in Figure 5E we were able to co-precipitate PSD95 together with LRP1, but observed no difference in the binding properties of PSD95 to LRP1 in LRP1ΔNPxY2 neurons versus wild type controls. However, a dephosphorylation of proteins in cell lysates of wild type neurons by CIP led to an increase in the amount of PSD95-protein co-precipitated with LRP1 (Figure 5F). Interestingly, a CIP treatment had no effect on the interaction of PSD95 with LRP1 in cell lysates of LRP1ΔNPxy2 neurons (Figure 5F lower panels). Taken together these results suggest a phosphorylation-dependent mechanism of LRP1 - NR2B subunit interaction, which might be linked by PSD95. However this mechanism is perturbed in the LRP1ΔNPxY2 *knock-in* background leading to a stronger binding of LRP1 with NR2B and an altered cell surface distribution of NR2B NMDA receptor subunit.

### Animals carrying the LRP1∆NPxY2 knock-in mutation demonstrate hyperactivity and impaired learning

**Figure 6:**
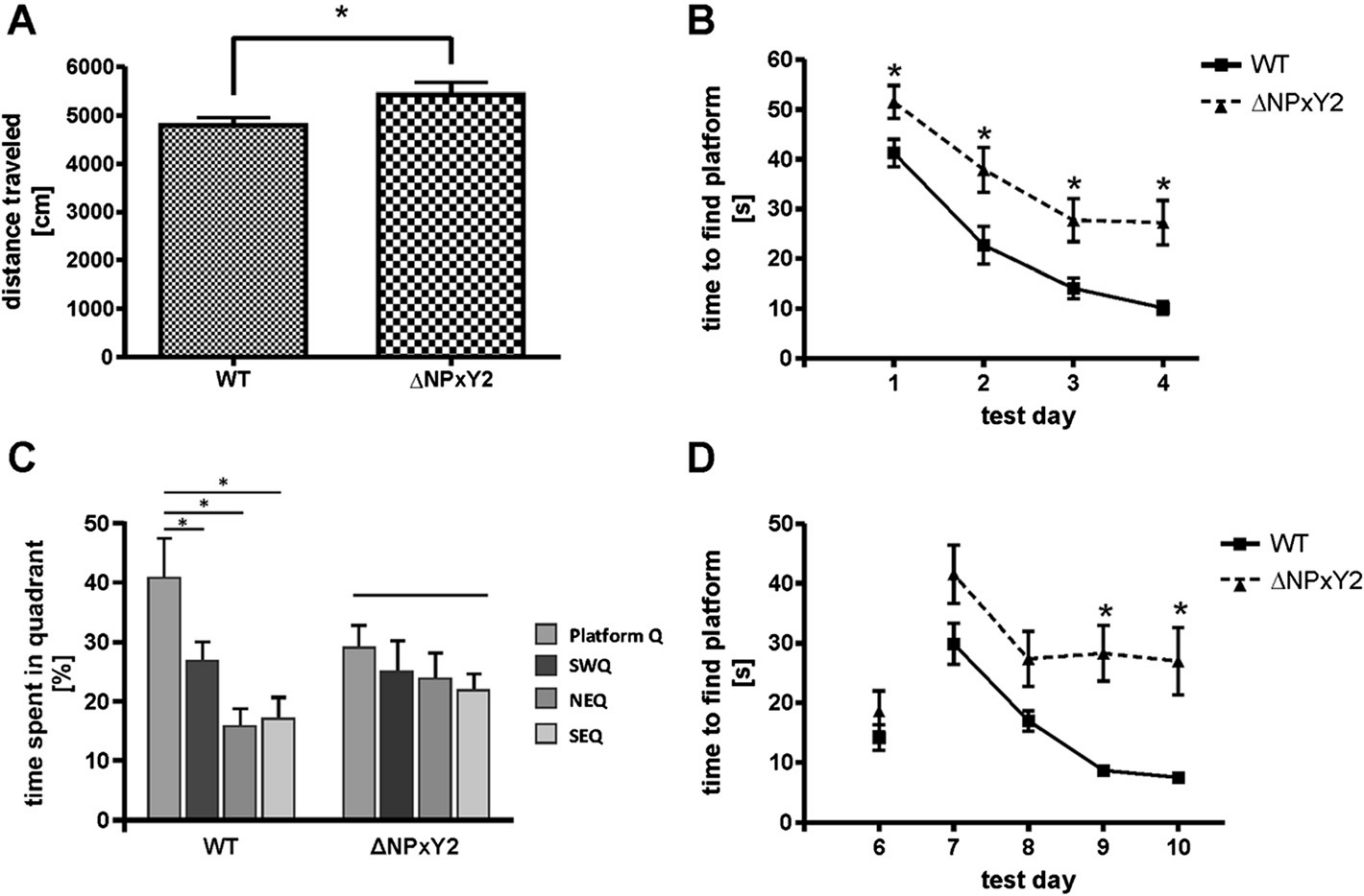
**LRP1**Δ**NPxY2 mice show hyperactivity and impaired learning behavior. (A)** Activity related analysis in an open field revealed a hyperactivity of LRP1ΔNPxY2 mice (n = 11) compared with WT littermates (n = 15). The diagram represents total distance traveled in open field after 10 min of monitoring ± S.E.M. and demonstrate a significant increase by 13.5% (F(1;24) = 5.060 p < 0.05) for LRP1ΔNPxY2 animals. **(B)** Learning in the Morris water maze. Test days 1 to 4 display standard spatial learning of the hidden platform task, MANOVA displayed significant effects over time (factor A; F_(3;22)_ = 33.849 p < 0.001) and with respect to genotype (factor B; F_(1;24)_ = 20.980 p < 0.001). Significant post-hoc effects of LRP1ΔNPxY2 mice (n = 11) compared with WT littermates (n = 15) are indicated by *. **(C)** Memory related behavior based on time mice were searching the platform in a respective quadrant of the maze at the probe trial (day 5) indicated a significant preference (paired t-test *p < 0.05) for the quadrant the platform was located before for WT but not LRP1ΔNPxY2 mice (SWQ southwest quadrant, NEQ northeast quadrant, SEQ southeast quadrant). **(D)** The sixth test day 24 days after spatial learning indicated both genotypes were able to remember the earlier platform location. Reversal learning abilities are displayed by time to find the platform hidden in a new location at days 7 to 10, MANOVA indicated significant effects over time (factor A; F_(3;22)_ = 11.147 p < 0.001) as well as with respect to genotype (factor B; F_(1;24)_ = 17.736 p < 0.001). Significant post-hoc effects of LRP1Δ NPxY2 mice (n = 11) compared with WT littermates (n = 15) are indicated by *.

Using our LRP1∆NPxY2 *knock-in* model we were able to demonstrate changes in NMDAR subunit distribution in cortical neuronal cultures. It has been shown by several groups, that correct NMDAR localization is a prerequisite for normal learning and memory behavior *in vivo*[18,33]. Additionally, it has been previously shown by other groups that a neuronal or a forebrain *knock-out* of the entire *Lrp1* gene result in an increased motor activity of the animals [26,27]. In contrast to the previously used *knock-out* of the entire *Lrp1* gene we used in our studies a *knock-in* mutation in the endogenous gene, which results in a functional *knock-out* of the NPxY2 motif. Therefore, we tested whether this limited *loss of function* mutation is sufficient to cause the behavioral changes associated with the neuronal *knock-out* of entire *Lrp1* gene reported earlier and investigated the voluntary motor activity of LRP1ΔNPxY2 animals in the open field paradigm [26,27]. As shown in Figure 6A the LRP1ΔNPxY2 animals demonstrated a significant 13.5% (F(1;24) = 5.060 p < 0.05) increase in voluntary motor activity in the open field paradigm, concomitant with earlier reported hyperactivity of animals harboring neuronal LRP1 *knock-out* or a forebrain *knock-out* mutation [26,27]. With respect to the MWM test for learning and memory (Figure 6B-D) no differences in activity between genotypes measured by swim speed were seen (F(1,24) = 1,687 n.s.) allowing for evaluation of cognitive performance. As shown in Figures 6B and 6D the animals harboring LRP1 *knock-in* mutation demonstrate significant changes in spatial and reversal learning in MWM paradigm compared with the wild type littermates. While in wild type mice time to reach the platform decreased from 41.3 s ±2.7 s to 10.2 s ±1.3 s in mean; the mice harboring LRP1 *knock-in* mutation needed at each of the four time points at least 27.2 s ±4.5 s (F(1;24) = 20.98; p < 0.001) to find the submerged platform. For the reversal learning a similar picture was found, wild type improved performance by decreasing time to find the new location of the platform (29.9 ± 3.4 s – 7.5 ± 0.9 s) while *knock-in* mice did not (41.5 ± 4.6 – 27.0 ± 5.6 s). Both behavioral aspects have been earlier demonstrated to be NMDA receptor signaling dependent [34-37]. The demonstrated behavioral changes indicate a physiological relevance of the observed alterations in NR2B surface expression on the NMDA receptor signaling in the neurons of LRP1ΔNPxY2 animals. In line with the observations on learning were the differences seen on day 5 for memory (Figure 6C). WT mice spent significantly more time (40.8 ±5.3%) in the quadrant where the platform has been compared to the other three and thus showing a significant preference for that quadrant compared to LRP1ΔNPxY2 mice (29.1 ±3.1%) which failed to show any preference.

Taken together, the presented results of biochemical and behavioral experiments demonstrate a critical role of LRP1 in internalization of NMDA receptor subunit NR2B and indicate a regulatory role of LRP1 NPxY2 motif in this process.

## Discussion

LRP1 has been frequently attributed to NMDA receptor function without addressing the exact molecular mechanism [7,8,26,27]. We have previously postulated a model for a functional role of LRP1 in tPa-mediated NMDA receptor signaling [8] and showed that a *knock-in* into the NPxY2 motif (LRP1ΔNPxY2) of LRP1 led to a reduction in NMDAR-mediated Erk1/2 activation. Furthermore, a direct effect of a neuronal LRP1 *knock-out* on the expression rates of NMDA receptor subunit NR1 has been recently reported [27]. Based on these results we now investigated the surface expression of LRP1 and NMDA receptor subunits in primary cortical neurons derived of LRP1ΔNPxY2 mice and observed an increase in LRP1 surface expression compared to control neurons (Figure 1A, B). Since the *knock-in* in the NPxY2 motif overlaps with YxxL endocytosis motif of LRP1, which is also affected by the alanine substitution, the reduction in neuronal endocytosis of LRP1 mimics the reduced internalization of LRP1 recorded already in other cell types [2,4,28]. Most interestingly, we observed an additional increase in the surface expression of the NMDA receptor subunits NR1 and NR2B, but not of NR2A in primary LRP1ΔNPxY2 neurons (Figure 1A). Although we were not able to demonstrate NR1 binding to LRP1, we were able to demonstrate by co-immunoprecipitation the binding of the NR2B subunit to LRP1 (Figure 5B). Based on these results we propose that the NR2B subunit binds to LRP1 and that the reduced endocytosis of the NR2B subunit in LRP1ΔNPxY2 neurons directly depends on the reduced LRP1 internalization. However, the increase in the NR1 expression at the cell surface in primary LRP1ΔNPxY2 neurons might be an indirect effect of LRP1 as further discussed below.

To further test this hypothesis we analyzed whether reduced protein translation might reveal differences in the overall protein degradation in primary LRP1ΔNPxY2 versus wild type neurons. We employed a cycloheximide (CHX)-mediated protein degradation assay. The CHX-treatment leads to a reduced protein translation rate in the cells and consequently reduces the available protein pool for the transport to the cell surface. Since the internalization and degradation of the surface proteins are not affected by CHX, an otherwise induced inhibition of the endocytosis would increase the residual protein amounts at the cell surface after prolonged CHX treatment. As expected, an increase in the residual expression of LRP1 and NR1/NR2B, but not of NR2A at the cell surface was observed in LRP1ΔNPxY2 neurons after CHX treatment for different time periods, while the degradation kinetics of the investigated proteins in the input controls are not altered (Figure 2). These data further highlight that the observed increase in NR1/NR2B receptor subunits might be a direct effect of the reduced internalization of LRP1 in LRP1ΔNPxY2 neurons.

To directly address this assumption we performed a cleavable NHS-SS biotin internalization assay and demonstrated reduced internalization rates of LRP1 and NR2B/NR1 receptor subunits in LRP1ΔNPxY2 neurons compared to internalization rates in wild type controls (Figure 3). Additionally we tested the possibility that the observed accumulation of LRP1 and NR1/NR2B at the cell surface of LRP1ΔNPxY2 neurons might be an apparent effect due to an increased recycling rate of these proteins on the cell surface. In this case we should observe an apparent increase in internalization rates in LRP1ΔNPxY2 neurons after a shorter period of internalization. The investigated shorter time period of 7 min revealed a significant reduction in internalization rates for LRP1 and NR1/NR2B but not for NR2A in LRP1ΔNPxY2 neurons compared to wild type controls (Figure 3C). These data confirmed our assumption that observed accumulation of LRP1 and NR1/NR2B at the surface of LRP1ΔNPxY2 neurons is an effect of reduced internalization of these receptor proteins due to a *knock-in* mutation in NPxY2 motif of LRP1.

We and others have previously shown that LRP1 might interact with the NR2B subunit and influence downstream signaling [8,26]. However, none of such interactions with LRP1 have been demonstrated for the NR1 subunit, which is transported to the cell surface solely as a part of functional heterotetramere formed in late Golgi [38,39]. According to these results we investigated the interaction of NR1 with LRP1 in co-immunoprecipitation experiments. As demonstrated in Figure 5A, we were not able to co-precipitate NR1 with LRP1 and consequently were not able to demonstrate a direct interaction of both proteins. Therefore, we concluded that only the NR2B receptor subunit is directly affected in the LRP1ΔNPxY2 *knock-in* background and the observed increase in NR1 surface deposition is rather a result of altered NR2B surface expression than an effect of different regulation of the NR1 subunit. Furthermore, the internalization rates for the NR1 and NR2B subunits are decreased in a similar ratio (by 69% and 70% respectively after 15 min and by 42% and 39% after 7 min) these data indicate that these subunits are included in the same functional NMDAR complex. We therefore postulate that LRP1 retains the NR2B subunit at the cell surface, which then retains the NR1 subunit in a complex.

We believe that we have presented convincing data, indicating that the surface up regulation of NR1 subunit is an effect of an increased NR2B subunit surface expression. These results are partly inconsistent with published results demonstrating a down regulation of NR1 receptor subunit in the brain homogenates of animals harboring a forebrain LRP1 *knock-out* mutation [27]. In contrast to this full LRP1 *knock-out* model, using our LRP1 *knock-in* mouse model we observed no alterations in the expression rates of the NR1 subunits in neuronal cell lysates (Figure 1) or in brain homogenates of LRP1ΔNPxY2 animals (data not shown) [27]. A possible explanation for this discrepancy might be the fact, that the alanine substitution in NPxY2 motif of LRP1 merely modifies the function of the protein, while a full *knock-out* of the entire *Lrp1* gene might affect additional regulatory pathways of the NR1 subunit expression [2,4,40]. Consistent with the data of Liu and colleagues, we observed no significant differences in the *steady state* expression rates of NR2B receptor subunit in primary neurons or brain homogenates derived from LRP1ΔNPxY2 animals (Figure 1A,B) [27]. These data indicate that the *knock-in* mutation in LRP1 rather affect the cellular distribution of the NR2B receptor subunit due to impaired internalization, than the general *steady state* expression of NR2B.

Recently a model for the regulation of the surface expression of NR2B has been proposed in which the coordinated phosphorylation of serine S1480 within the PDZ-domain binding motif ESDV and of tyrosine Y1472 within the internalization motif YEKL have been demonstrated to regulate the NR2B receptor endocytosis [21]. According to this model an increased phosphorylation on S1480 and a decreased phosphorylation on Y1472 might function as a signal for enhanced endocytosis of NR2B receptor. Therefore we hypothesized that the alterations in the phosphorylation state of these regulatory signals might be a possible mechanistic explanation for the demonstrated increase in surface expression and a lower internalization rate of the NR2B subunits in LRP1ΔNPxY2 neurons. Most interestingly, we observed an increase of S1480 phosphorylation and a decrease of phosphorylation of Y1472 of NR2B at the cell surface of LRP1ΔNPxY2 neurons (Figure 4). Based on previous publications one would assume that this phosphorylation pattern should lead to an increased endocytosis rate of the NR2B receptor. However in LRP1ΔNPxY2 neurons we see a reduced endocytosis despite the enhanced activation of these signals compared to the wild type controls. We investigated the expression and activation rates of responsible kinases to exclude a dysregulation of kinases involved in NMDAR phosphorylation as a possible explanation for the altered phosphorylation pattern of the NR2B subunit in LRP1ΔNPxY2 neurons. However, we were unable to detect any differences in activity or expression of respective kinases in LRP1ΔNPxY2 neurons (Figure 4C). Therefore, these data indicate that the CKII or Fyn kinases signaling is not affected by LRP1ΔNPxY2 mutation and the observed increase in NR2B surface expression might rather be a direct effect of reduced LRP1 internalization rate in LRP1ΔNPxY2 neurons.

Although the NR2B subunits are strongly phosphorylated in neurons derived of LRP1ΔNPxY2 mice the accumulation of NR2B subunits at the cell surface seems to overcome the regulation by CKII and Fyn signaling. Therefore, we speculate that LRP1 holds the NMDA receptor subunits at the cell surface although the internalization signals of NR2B are still activated. We were able to demonstrate a stronger interaction of NR2B receptor subunits to LRP1 in neurons derived from LRP1ΔNPxY2 animals compared to the wild type controls, most likely due to increased interaction duration by reduced endocytosis (Figure 5B). Next we asked whether the phosphorylation pattern of the proteins might influence the binding of NR2B with LRP1. We addressed this question using a Calf Intestinal Phosphatase (CIP) dephosphorylation assay; and analyzed whether a dephosphorylation of the proteins might influence the binding properties of the NR2B subunit to wild type or mutated LRP1. As shown in Figure 5D CIP treatment drastically reduced the amounts of NR2B co-immunoprecipitated with LRP1 in wild type neuronal lysates. These data demonstrate an interruption of the binding of the NR2B subunit to wild type LRP1 after dephosphorylation, while the binding of the NR2B subunit to LRP1ΔNPxY2 is not affected. Furthermore we addressed a possible role of PSD95 as a linker between the NR2B subunit and LRP1. The binding of LRP1 and PSD95 demonstrated in Figure 5E and the earlier reported role of PSD95 in tPa-mediated LRP1-NMDAR signaling indicate PSD95 as a possible linker between these proteins [8]. As shown in Figure 5E we were not able to demonstrate any differences in the binding strength of PSD95 to LRP1 between neuronal lysates of LRP1ΔNPxY2 neurons or wild type controls. This result indicates that the NPxY2 motif of LRP1 is probably not the sole binding site for PSD95. Interestingly, we were able to demonstrate a stronger binding of PSD95 to LRP1 after dephosphorylation in lysates derived from wild type neurons, while a dephosphorylation of proteins in lysates derived from LRP1ΔNPxY2 neurons did not show altered binding of PSD95 with LRP1 (Figure 5F). Evidently, a dephosphorylation of the NPxY2 motif of LRP1 enhances the binding of PSD95 in wild type neurons. This observation indicates that either the NPxY2 motif in a dephosphorylated state might bind PSD95 or the phosphorylation of the NPxY2 motif might regulate the binding of PSD95 to another position in the LRP1 C-terminus. We speculate, due to the lack of the functional NPxY2 motif, tyrosine phosphorylation is impaired in the LRP1ΔNPxY2 *knock-in* background. This might lead to a dysregulation of the phosphorylation dependent binding of PSD95 with LRP1. We propose that an increased PSD95-LRP1 interaction might impair the interaction of the NR2B subunit with LRP1 since we observe the inverse binding capacity of the NR2B subunit to LRP1 after CIP treatment compared to PSD95 (compare upper panels in Figure 5D to 5F). Moreover, it has been shown that an initial tyrosine phosphorylation of the NPxY2 motif is a prerequisite for the phosphorylation of the NPxY1 motif in the C-terminus of LRP1 and increases its accessibility for interaction partners [5,6]. Therefore we hypothesize that dephosphorylation of LRP1 leads normally to a disruption of the LRP1-NR2B binding due to conformational changes within the C-terminal part of LRP1. Since the second NPxY motif in *knock-in* LRP1ΔNPxY2 neurons cannot be phosphorylated the receptors might stick together, which results in the retention of NR2B at the cell surface. To summarize this part, we propose a mechanism in which the NR2B receptor subunit is internalized in a LRP1-dependent manner. The interaction of the proteins seems to be phosphorylation dependent. The control of the interaction requires an intact NPxY2 motif of LRP1. The interaction might be linked by PSD95 but its exact role is not clear.

The alterations in NMDA surface expression lead to deficits in LTP/LTD induction and memory formation [18,33]. Furthermore, a neuronal or a forebrain LRP1 *knock-out* result in NMDA receptor signaling-associated behavioral changes of animals harboring the mutation [26,27]. Therefore, we tested whether the *knock-in* into NPxY2 motif of LRP1 is sufficient to induce a similar phenotype as reported for neuronal or forebrain *knock-out* of the entire *Lrp1* gene [26,27]. As demonstrated in Figure 6A LRP1ΔNPxY2 animals display an increased activity in open field paradigm, earlier reported for neuronal LRP1 *knock-out* and forebrain LRP1 *knock-out* animals [26,27]. Referring to the described hyperactivity in response to NMDA-antagonists treatment, these results indicate an impaired signaling of NMDA receptors in LRP1ΔNPxY2 animals [41,42]. Over expression of the NR2B subunit in forebrain of transgenic mice led to a superior memory in a number of behavioral tasks [43]. Additionally, a line of evidences indicating a superior role of NR2B subunit containing NMDA receptors in learning was already published [44-49]. Therefore, we expanded the behavioral characterization of the LRP1ΔNPxY2 mice to NMDA-associated behavioral tasks like spatial and reversal learning [36,37]. However, as shown in Figure 6 the LRP1ΔNPxY2 animals have significant deficits in spatial and reversal learning and memory in Morris Water Maze task. While latencies (s) of trial 1 were not significantly different (45.0 ± 4.7 vs. 52.2 ± 4.1) the combination of all four trials of the first training day already showed an impairment of LRP1ΔNPxY2 animals. These results indicate behavioral changes similar to those associated with impaired NMDA receptor signaling [34,35]. The present phenotype also corresponds to observations implicating reversal learning in behavioral flexibility, a neuronal feature also related to plasticity mechanisms like LTP and LTD. The later one however, has been discussed in light of NR2B related mechanisms and behavioral flexibility [44-46]. Moreover, seen from this aspect, the observed deficits in reversal learning are at least in part consistent with deficits in LTP-induction in brain slices of LRP1 forebrain *knock-out* animals demonstrated by Liu and colleagues [27]. Thus, behavioral results allude that a functional *knock-out* of the NPxY2 motif of LRP1 to be sufficient to induce behavioral changes reported for neuronal and forebrain *knock-out* of entire *Lrp1* gene and thereby indicating a differential role of the NPxY2 motif of LRP1 both on internalization and signaling of the NMDA receptor [26,27].

## Conclusions

In summary, our study suggests a model, in which the functional interaction of LRP1 and NR2B requires an intact NPxY2 motif of LRP1 and is regulated by phosphorylation. In our model a substitution of LRP1 NPxY2 motif by alanines led to a stronger binding of NR2B receptor subunit to LRP1 and a decoupling of the surface expression of NR2B from regulation by CKII and Fyn kinases.

## Materials and methods

### Animals

C57Bl6 wild-type (WT) or C57Bl6 LRP1 NPxYxxL *knock-in* mice harboring the inactivated mutant NPxYxxL motif as previously described [3] were used for behavioral studies and for the isolation of primary cortical neurons. Animals were housed three to four per cage (810 cm^2^, type III) at 22°C and 60% relative humidity. Food and water were provided ad libitum and a 12 h light–dark cycle was maintained. For the behavioral studies, 26 male age-matched mice were tested from 3 month onward. All experimental procedures were carried out in accordance with the European Communities Council Directive regarding care and use of animals for experimental procedures and were approved by Landesuntersuchungsamt Koblenz, Germany.

### Antibodies

The following primary antibodies were used: mouse monoclonal anti-NMDA receptor subunit NR2B antibody and rabbit polyclonal anti-phospho NR2B (NR2BpSer1480) (Thermo Fisher Scientific), rabbit polyclonal anti-phospho NR2B (NR2BpTyr1472) (Millipore), mouse monoclonal anti-NMDA receptor subunit NR1 (Zymed), rabbit polyclonal anti-NMDA receptor subunit NR2A (Santa Cruz Biotechnology), rabbit polyclonal LRP1 antibody 1704 [50], rabbit polyclonal anti-phospho-LRP1 antibody pLRP1Y4507 (Santa Cruz Biotechnology), rabbit polyclonal anti-actin (Sigma-Aldrich), rabbit polyclonal anti-phospho-Src [pY418] (Invitrogen); rabbit monoclonal anti-PSD95 (clone EP2652Y, Millipore); mouse monoclonal anti-Fyn clone 25/Fyn (B&D Transduction Laboratories); rabbit polyclonal anti-Casein Kinase CKII (Upstate). Secondary horseradish peroxidase-conjugated antibodies: goat polyclonal anti-rabbit (Sigma-Aldrich) and donkey polyclonal anti-mouse (Jackson Laboratories) were used.

### Chemicals

Sulfo-NHS-LC-LC-Biotin and Sulfo-NHS-SS-Biotin were both purchased from Pierce (Thermo Fisher Scientific). Sodium 2-mercaptoethane sulfonate (MesNa), iodacetamide (IAA) and cycloheximide (CHX) were purchased from Sigma-Aldrich. Calf intestinal phosphatase (CIP) was purchased from New England Biolabs.

### Cell culture

Primary cortical neurons were isolated from mouse embryos at embryonic day 16. In brief, cortices were collected in ice cold Hank’s balanced saline solution (HBSS, Invitrogen). After trypsinization (0.05% trypsin, 0.02% EDTA in phosphate-buffered saline) at room temperature for 20 min tissue was centrifuged at 300 × g for 2 min. Cells were resuspended in Neurobasal medium (NBS, Invitrogen) containing B-27 supplement and 1xGlutamax (both Invitrogen) and mechanically dissociated by pipetting. Cells were filtered through a nylon mesh and centrifuged at 300 × g for 5 min. 6.5×10^5^ cells were plated on poly-L-ornithine (100 μg/ml, Sigma-Aldrich) coated 6-cm dishes and cultured at 37°C in a humidified 5% CO2 incubator for 14 days.

### SDS-PAGE, western blot analysis and immunoprecipitations

Whole brains of LRP1ΔNPxY2 or wild type animals were dissected and immediately frozen in liquid nitrogen. Afterwards the brain tissue was weighed and homogenized in 6fold volume (w/v) of radioimmune precipitation buffer RIPA (RIPA: 50 mM Tris–HCl pH 8.0; 1% Nonident P-40; 0.5% deoxycholate; 0.1% SDS) containing protease inhibitor cocktail (complete, Roche Applied Sciences), phosphatase inhibitor cocktail (PhosSTOP^®^, Roche Applied Science) and 1 mM sodium orthovanadate (Sigma-Aldrich) in a dounce homogenizer. The obtained homogenate was incubated on ice for 30 min and centrifuged in an ultracentrifuge (Beckman) at 55.000 rpm at 4°C for 30 min. The supernatants were collected and protein concentrations were determined by BCA protein assay (Pierce). Equal amounts of proteins (20 μg) were loaded on SDS-PAGE gels and analyzed by western blot technique using appropriative antibodies.

Primary cortical neurons were lysed in radioimmune precipitation buffer (RIPA: 50 mM Tris–HCl pH 8.0; 1% Nonident P-40; 0.5% deoxycholate; 0.1% SDS) containing protease inhibitor cocktail (complete, Roche Applied Sciences), phosphatase inhibitor cocktail (PhosSTOP^®^, Roche Applied Science) and 1 mM sodium orthovanadate (Sigma-Aldrich). Protein concentrations were determined by BCA protein assay (Pierce) and equal amounts of total proteins were precipitated with the appropriate primary antibodies and protein G sepharose (GE Healthcare) at 4°C overnight. Beads were collected by centrifugation, eluted in 2xSDS sample buffer and boiled at 95°C for 5 min. Proteins were separated on 10% Tris-glycine gels under reducing conditions and transferred to a nitrocellulose membrane (Whatman). The membrane was blocked with Tris-buffered saline containing 0.1% Tween 20 (Roth) and either 5% milk or 3% BSA (Sigma) (the latter for phospho-specific antibodies). Incubation with primary antibodies was carried out at 4°C overnight. Afterwards the blots were incubated with the appropriate secondary antibodies. The protein bands were visualized by ECL reagent (Millipore), using the LAS-3000 mini (Fujifilm).

### Dephosphorylation of proteins with calf intestinal phosphatase (CIP)

For the co-immunoprecipitation experiments with LRP1 and NR2B the cell lysates were treated with Calf Intestinal Phosphatase (CIP, NEB). In brief, the neurons were lysed in RIPA buffer containing protease inhibitors and protein concentration was determined. Afterwards 100 μg of proteins were treated with 1000 Units of CIP in the phosphatase buffer (50 mM Tris–HCl; 100 mM NaCl; 10 mM MgCl_2_; 1 mM Dithiotreitol, pH 7.9) at 37°C for 30 min. The dephosphorylated lysate was further analyzed by immunoprecipitation and western blotting.

### Cell surface biotinylation

The following experiments were carried out with cortical neurons at DIV14. For the cell surface biotinylation, cells were washed 3 times with ice cold phosphate-buffered saline (PBS) and incubated with 2.5 mg/ml Sulfo-NHS-LC-LC-Biotin in PBS at 4°C for 40 min preventing internalization. Afterwards, the unconjugated biotin was quenched with 50 mM NH_4_Cl in PBS and the cells were scraped off the plates and collected in ice cold PBS. After cell lysis and protein concentration determination, equal amounts of proteins were incubated with NeutrAvidin agarose beads (Thermo Fisher Scientific) at 4°C overnight. Biotinylated proteins were eluted by boiling at 95°C in 2xSDS sample buffer for 5 min and separated on 10% Tris-glycine gels.

### Cleavable biotin internalization assay

The internalization rates of the proteins were determined employing the biotin internalization assay. In brief, the neurons were surface labeled with 2.5 mg/ml cleavable Sulfo-NHS-SS-Biotin (Thermo Fisher Scientific) in cold PBS at 4°C for 30 min. Cells were then rinsed with warm NBS medium and incubated at 37°C for 7 or 15 min allowing internalization. Afterwards the neurons were incubated in ice cold stripping buffer (20 mM MesNa in 50 mM Tris–HCl, pH 8.6; 100 mM NaCl in ddH_2_O) at 4°C for 20 min. The stripping solution was quenched by incubation in 20 mM iodoacetamide (Sigma) in ice cold PBS at 4°C for 10 min. Neurons were then lysed in RIPA buffer containing protease inhibitor cocktail (Roche Applied Science). The equal amounts of protein were incubated with NeutrAvidin agarose (Thermo Fisher Scientific) at 4°C overnight. Isolated proteins were recovered by adding 2xSDS sample buffer and boiling at 95°C for 5 min and separated on 10% Tris-Glycine gels.

### Protein degradation assay

Primary neurons at DIV14 were treated with 20 μg/ml cycloheximide at 37°C. At defined time points, cells were biotinylated and the protein expression at the cell surface or in cell lysates was analyzed by SDS-PAGE and Western blot.

### Behavioral testing

The sequence of testing was performed in the following order: D*ay 1*: open field to measure general activity and exploration. *Days 6–9:* Morris water maze test to measure spatial learning. *Day 10:* Morris water maze test to measure memory performance in a probe trial without platform. *Days 34–38:* Morris water maze test reversal learning.

### Open field activity

General activity of mice was assessed during a 10 min session in an open field paradigm. The test arena measured 60 × 60 × 40 cm. The parameters recorded during each trial were: total distance traveled (cm), resting time (% of total recording time not moving), time spent along the walled parts of the maze (10 cm corridor; % of total time) and entries into the central part of the maze (30 cm diameter; number, n) [51,52].

### Morris water maze

Spatial learning and memory were tested by the Morris water maze hidden platform task using the same maze and protocol as described [52,53]. The platform stayed in the same quadrant for days 1 to four and the animals were released from four different positions at the pool perimeter. Mice performed four trials per day on four consecutive days with a maximum length of 60 s and an inter-trial interval of 90 s. If mice did not find the platform within the given time they were gently guided to the platform. Mice were allowed to stay on the platform for 10 s. On the fifth water maze day, a probe trial (60 s) without platform was performed. Learning was assessed by measuring the latency to find the platform and the distance swum. General activity was assessed by swim speed and memory capabilities were characterized by the time each mouse spent in each quadrant searching for the platform at probe trial. For evaluation of reversal learning 24 days after spatial learning tests mice were given one day (day 6) with four trials with the former platform location and gentle guidance as reported before. Starting at day 7 to 10 (four trials each) platform location was changed to an opposite position and animals were removed from the pool when the platform was not found within the 60 sec time frame.

### Monitoring of behavior

A computerized video system registered moving-path and duration in open field and water maze tests automatically. The hardware consisted of an IBM-type AT computer combined with a video digitizer and a CCD video camera. The software used for data acquisition and analysis was EthoVision XT^®^ release 8.0 (Noldus Information Technology, Utrecht, Netherlands).

### Quantification and statistical analysis

Western blots were quantified by densitometry using Image J 1.44. All graphs and statistical analysis were prepared using GraphPad Prism 4 software (La Jolla, CA). Data were analyzed by one-way analysis of variance (ANOVA) coupled Newman-Keuls test for multiple comparison or *t* test. p < 0.05 was considered as statistically significant.

For the behavioral studies, data were analyzed by ANOVA for differences between the genotypes. Multivariate analysis of variance (2-way ANOVA) was performed for learning and memory testing. Probe trial scores within experimental groups were evaluated by paired t-test. Differences were considered as significant for p ≤ 0.05.

## Abbreviations

NMDA: N-methyl-D-aspartate; NR1: NMDA receptor subunit 1; NR2A: NMDA receptor subunit 2A; NR2B: NMDA receptor subunit 2B; LRP1: Low density lipoprotein receptor-related protein 1; NPxY2: Distal NPxY motif; LRP1ΔNPxY2: LRP1 with alanine substituted NPxY2 motif; CKII: Casein kinase II; CIP: Calf intestinal phosphatase; CHX: Cycloheximide; tPa tissue-type: plasminogen activator; PSD95: Post synaptic density 95.

## Competing interests

The authors declare no competing financial interests.

## Authors’ contributions

WM has designed and carried out the biochemical experiments, prepared and wrote the manuscript. MB has carried out the behavioral studies. SM has contributed to the biochemical experiments and the writing of the manuscript. AR has provided the LRP1ΔNPxY2 animals and contributed to the proof readings of the manuscript. SW has contributed to the experimental design and the writing of the manuscript. US has supervised, designed and interpreted the behavioral studies and contributed to the writing of the manuscript. CUP has supervised the experimental design and entire work at the manuscript. All authors read and approved the final manuscript.

## Supplementary Material

Additional file 1: Figure S1 Primary LRP1ΔNPxY2 neurons demonstrate any significant alterations in cell viability or neurite outgrowth. (**A**) LRP1ΔNPxY2 neurons demonstrate any significant alterations of cell viability in alamarBlue reduction assay. The alamarBlue cell viability assay was performed as described in Methods section. For the measurement three 6-well plates for each genotype were used. The presented 562 nm/590 nm ratio reflects the percentage of reduced alamarBlue after 4 h minus the absorbance of alamarBlue in the no-cell blank controls. The scale bars represent mean percent of reduced alamarBlue reagent after 4 h + S.E.M. for the wild type controls 13.9% and for LRP1ΔNPxY2 neurons 13% (n = 3 6well plates for each genotype). (**B**) LRP1ΔNPxY2 neurons demonstrate no alterations in neurite outgrowth compared to the wild type controls. The primary cortical neurons were isolated from LRP1ΔNPxY2 or control animals and cultivated for 3 days. After an immunostaining with a specific MAP-2 antibody the neurite length was analyzed using LSM-710 microscope and ZEN software (Zeiss). The mean neurite length was calculated as the ratio of total neurite length to the number of neurons analyzed. The scale bars represent the mean of total neurite length in μm + S.E.M. For wild type controls 24.2 μm and for LRP1ΔNPxY2 neurons 25.8 μm (both n = 100). Click here for file

Additional file 2 Materials and Methods. Click here for file
