# Supplementary material for: LRP1 is critical for the surface distribution and internalization of the NR2B NMDA receptor subtype
Source: Mol Neurodegener. 2013 Jul 17;8:25. doi: 10.1186/1750-1326-8-25 (PMC3722104; doi:10.1186/1750-1326-8-25)
Supplement: Additional file 2 — Materials and Methods. [file 1750-1326-8-25-S2.docx]

**Supplementary**

**Materials and Methods**

*AlamarBlue cell viability Assay*

Primary neurons derived from LRP1ΔNPxY2 or wild type animals were seeded on a 6-well plate at a density of 1.5 x 10^6^ cells for the entire plate. After cultivation for 5 days in an incubator at 37°C and 5% CO_2_ the medium was removed and replaced with 1ml fresh NBS containing 10% of alamarBlue reagent (Invitrogen) for each well according to the manufacturer’s protocol. Afterwards the plates were incubated at 37°C and 5% CO_2_ for 4 hours. Two hundred micro liters of alamarBlue-containing medium was removed from the well and pipetted into a 96-well plate for reading. The absorbance of reduced alamarBlue was read at 562nm and the absorbance of oxidized alamarBlue was read at 595nm with a 96-well plate reader (Anthos 2010; Microsysteme, Germany). The presented 562nm/590nm ratio reflects the percentage of reduced alamarBlue after 4h minus the absorbance of alamarBlue in the no-cell blank controls. Three 6-well plates for each genotype were analyzed.

*Measuring of neurite outgrowth*

For the measurement of neurite outgrowth the primary neurons were seeded on poly-ornithine coated cover slip in a 12-well plate at a density of 1x 10^6^ cells for entire well. At DIV3 the cells were fixed with 4% paraformaldehyde in PBS for 10 min, permeabilized with 0.1% Triton-X in PBS for 20 min and blocked with 4% BSA for 1h. Afterwards the cells were incubated with primary monoclonal MAP-2 antibody (Sigma Aldrich) diluted 1:300 in 4 % BSA solution at 4°C overnight. As secondary antibody a goat-anti-mouse Alexa-flour-488 conjugated antibody (Invitrogen) was used. The cells were visualized with a laser-scanning microscope LSM-710 (Zeiss, Jena) and the length of neurite outgrowth was analyzed using ZEN software (Zeiss). Five cover slips and four randomly selected fields were selected five cells on each filed for LRP1ΔNPxY2 or wild type controls were analyzed. The mean neurite length was calculated as the ratio of total neurite length to the number of neurons analyzed.

**Figure legends**

Suppl. Figure 1. Primary LRP1ΔNPxY2 neurons demonstrate any significant alterations in cell viability or neurite outgrowth.

(A) LRP1ΔNPxY2 neurons demonstrate any significant alterations of cell viability in alamarBlue reduction assay. The alamarBlue cell viability assay was performed as described in Methods section. For the measurement three 6-well plates for each genotype were used. The presented 562nm/590nm ratio reflects the percentage of reduced alamarBlue after 4h minus the absorbance of alamarBlue in the no-cell blank controls. The scale bars represent mean percent of reduced alamarBlue reagent after 4h + S.E.M. for the wild type controls 13.9% and for LRP1ΔNPxY2 neurons 13% (n=3 6well plates for each genotype).

(B) LRP1ΔNPxY2 neurons demonstrate no alterations in neurite outgrowth compared to the wild type controls. The primary cortical neurons were isolated from LRP1ΔNPxY2 or control animals and cultivated for 3 days. After an immunostaining with a specific MAP-2 antibody the neurite length was analyzed using LSM-710 microscope and ZEN software (Zeiss). The mean neurite length was calculated as the ratio of total neurite length to the number of neurons analyzed. The scale bars represent the mean of total neurite length in µm + S.E.M. For wild type controls 24.2 µm and for LRP1ΔNPxY2 neurons 25.8 µm (both n=100).
